# Women’s Awareness of Reproductive Health

**DOI:** 10.3390/medicina60010158

**Published:** 2024-01-15

**Authors:** Oliwia Zalewska, Katarzyna Wszołek, Małgorzata Pięt, Maciej Wilczak, Karolina Chmaj-Wierzchowska

**Affiliations:** 1 Specialized Health Care Center for Mother and Child in Poznan, Obstetrics and Gynecology Department, 60-235 Poznan, Poland; oliwiazalewska68@gmail.com; 2 Department of Maternal and Child Health, Poznan University of Medical Sciences, 60-701 Poznan, Poland; mwil@ump.edu.pl (M.W.); karolinachmaj@poczta.onet.pl (K.C.-W.); 3 Facility of Practical Midwifery Study, Poznan University of Medical Sciences, 60-701 Poznan, Poland; mpiet@vp.pl

**Keywords:** reproductive health, women, knowledge, infertility

## Abstract

*Background and Objectives*: reproductive disorders are a serious global concern in medical, social, and demographic contexts. According to estimates, approximately 10–15% of couples around the world suffer from infertility. Numerous studies have shown that modifiable lifestyle factors, such as a high-fat diet, a postponed decision to start a family, tobacco smoking, alcohol consumption, risky sexual behavior, psychiatric diseases, and chronic stress, have a negative influence on the fertility of women. The main goal of this study is to assess the knowledge of women about reproductive health, infertility risk factors, and causes of infertility and to determine whether the level of this knowledge varies based on sociodemographic variables. *Materials and Methods*: a survey was conducted among 111 patients who anonymously filled in a questionnaire comprising questions regarding fertility and its deficiencies. The results were analyzed using the Chi-square test and Fisher’s test. *Results*: the survey results indicated that women had a good or very good level of knowledge of the causes of infertility. The obtained test results were statistically significant (*p* < 0.05), but the studied group did not possess sufficient knowledge of the symptoms characterizing the diseases related to limited fertility (*p* > 0.05). The level of knowledge on the diagnosis of infertility did not depend on the age of the examined people, their educational level, or personal experience in this field (*p* > 0.05). The results also revealed that the awareness of women on reproductive health was poor. The studied women had a low level of knowledge of infertility risk factors, and their knowledge did not correlate with age, educational level, or personal experiences. *Conclusions*: information on the aspects of reproductive health should be widely disseminated through public educational campaigns, aimed at correcting erroneous convictions among women about the risk factors for infertility and assisting them in improving fertility.

## 1. Introduction

Infertility has become a global health problem. According to the World Health Organization, approximately 10–15% of couples around the world are affected by this condition. The number of couples requiring diagnostics and treatment for fertility-related issues has been increasing every year [1,2]. The most common types of infertility in women are hormonal, ovarian, fallopian tubal, uterine, cervical, and immunologic infertility, infection-based infertility, and psychiatric and sexual disorder-related infertility [1]. Male infertility can be mostly caused by endocrine disorders, sperm transport disorders, and primary testicular defects or be idiopathic [3]. The risk of infertility can be increased by tobacco smoking, the overuse of alcohol and drugs, inadequate nutrition, an abnormal body mass index (<18.5 or >24.99 kg/m^2^), nutritional disorders, exposure to stress, and harmful sexual practices [4,5].

An excessive body weight is often associated with a high insulin level, which contributes to the development of insulin resistance and subsequently adipose tissue accumulation, polycystic ovary syndrome, and metabolic syndrome and intensifies the biosynthesis of androgens and lipids from cells [6,7]. In addition, irrational weight loss has been increasingly noticed among young women in recent years, which may lead to the development of severe mental diseases, such as anorexia and bulimia [8,9]. The risk of sexually transmitted diseases, such as gonococcal disease, chlamydia trachomatis, herpes, syphilis, and Human Immunodeficiency Virus (HIV), is increased by hazardous sexual practices. The lack of the use of contraceptives in suitable cases may lead to sexually transmitted infections and unplanned pregnancies, and thus abortions, which, if improperly conducted, may result in reproductive problems in the future [10]. It is well known that female fertility decreases with age. It has also been shown that the quality of ova deteriorates with age, which contributes to ovulation deficiency, reduced ovulation frequency, and an incompetent luteal phase, leading to decreased fertilization rates [1,5]. Furthermore, the incidence of genetic defects and spontaneous abortions increases with the mother’s age.

All of the above-mentioned risk factors should be known to young persons, not only girls and women. The awareness of health issues depends on many factors; however, education in the family environment and at school seem to be the most important [11]. We see the need to further explore the association of infertility with women’s awareness of reproductive health.

Considering that the majority of infertility risk factors are modifiable, primary prevention through increased awareness of the causes of infertility is of key importance [10]. Educational interventions should thus be directed at expanding the knowledge of couples willing to have offspring and providing them with the real picture of the infertility problem. More emphasis should be placed on the education of women when they disclose their difficulties in achieving pregnancy to their physician to improve their knowledge and change their attitude toward infertility [12]. Patient education appears to be one of the key factors shaping health-promoting attitudes, particularly in an era when doubtful information may be found on the internet. This medium can be used, as it is readily available, but skilled healthcare providers must develop webinars, podcasts, or educational videos. Video interventions have been effective in modifying other health behaviors of people, such as breast self-examination, screening tests for prostate cancer, and HIV testing [13]. Educational videos posted on social media can quickly reach many young adults [14]. However, it is important to pay attention to the content that is transferred in predominant models of sexual education.

The objective of this study was to verify the level of women’s knowledge about reproductive health, the risk factors of infertility, and the causes of procreation-related problems among women.

## 2. Materials and Methods

An anonymous survey, paper, and pencil questionnaire was conducted among women hospitalized in the Gynecological and Obstetrical Clinical Hospital of the Poznan University of Medical Sciences. The initial diagnosis was “infertility”—patients had been hospitalized for difficulty in getting pregnant and were awaiting diagnosis and treatment. A total of 111 patients agreed to fill out the questionnaire. The survey was conducted between October 2020 and May 2021.

The questionnaire included 22 questions. For all the questions, there was an option of choosing more than one answer. The first part of the questionnaire had questions related to general demographic information, while the second part consisted of questions on infertility and reproductive health. Women participated in a survey to gauge their comprehension of the definition of infertility. The study explored the interdependence of a woman’s fertility on age and various lifestyle factors. Additionally, it delved into the diseases that can lead to female infertility, emphasizing the significance of pathogenic factors in contributing to challenges in conception. The research also investigated the diagnostic tests conducted to identify and diagnose infertility issues. Furthermore, it highlighted the impact of mental health on the successful fertilization process, underlining the role it plays in overall reproductive health. Lastly, the study underscored the importance of psychological and psychiatric treatment in both the diagnostic and treatment phases of infertility, recognizing their potential contributions to a comprehensive and effective approach.

Patients completed a self-designed, non-validated survey questionnaire.

Statistical analysis was performed in PQStat software (ver. 1.6.8). A significance level of *p* < 0.05 was accepted. The analysis was based on the assessment of responses to the questions regarding the causes of infertility and knowledge of the symptoms characterizing the diseases related to limited fertility. The calculations assumed that at least 50% of correct answers indicated a sufficient level of knowledge. We considered such a level of knowledge to be minimal, and below a value of 50%, there can be no question of knowledge at a sufficient level. The Chi-square test was used to verify for one sample; it was verified whether the majority of the surveyed people had adequate knowledge. It also tested whether the level of knowledge depends on sociodemographic variables, such as age, education, and personal experience in treating infertility. The dependence of knowledge on statistical correlations between qualitative variables was evaluated using Fisher’s test.

## 3. Results

The characteristics of the study group are presented in Table 1.

The responses of the respondents to individual questions are presented in Table 2.

Among the 111 women surveyed, 39.6% had insufficient knowledge about the causes of infertility. On the other hand, 36.9% and 11.7% had rather good and good knowledge on factors causing infertility, while 6.3% and 5.4% exhibited more than good and very good levels of knowledge, respectively (*χ*^2^ = 4.765, *p* = 0.029). This suggests that the level of knowledge on the causes of infertility was satisfactory among the surveyed women (Table 3).

Over half of the respondents (58.6%) had insufficient knowledge on the symptoms accompanying the diseases related to limited fertility, while 32.4% of the surveyed exhibited rather good, 8.1% exhibited good, and 0.9% exhibited very good knowledge on this topic (*χ*^2^ = 3.252, *p* = 0.071). This indicates that the level of knowledge on the symptoms characterizing the diseases limiting fertility was unsatisfactory among the surveyed individuals (Table 4).

The results of Fisher’s test showed that the knowledge level did not depend on the age of the surveyed women (*p* > 0.05). The majority of them were aged less than 25 years (64.6%) and above 35 years (65.5%), some were between 26 and 35 years (44.1%), and all respondents exhibited an insufficient level of knowledge. The respondents’ level of knowledge on infertility diagnosis also did not depend on their educational level (*p* > 0.05). About 80% of people with primary or professional education, 65.3% with secondary education, and 44.7% with tertiary education had an insufficient level of knowledge. Furthermore, the analysis investigated whether women who had already been treated for infertility will possess a higher level of knowledge on this issue. However, regardless of personal experience, most of the respondents who had been treated earlier for fertility (45%) and those who had not been treated (61.5%) demonstrated an insufficient level of knowledge on infertility (*p* > 0.05).

## 4. Discussion

One of the most significant demographic tendencies observed during 1950–2017 was a marked decrease in the fertility rate [15]. Awareness of the risk factors for infertility, including the causes for its development, constitutes a significant step toward maintaining fertility by adopting lifestyle changes. An appropriate understanding of the definition of fertility and the age threshold after which reproductive capability starts to decrease will enable couples to plan a family at the right time and also aid them in maintaining a healthy pregnancy and preventing genetic defects in the fetus [16].

In our study, over half of the surveyed participants (54.1%) were aware that infertility refers to the inability to conceive after one year of regular intercourse without the use of contraceptives. A similar result (49%) was obtained by Bennett et al. [17]; however, in the study of Ali et al. [18], only 25% of participants believed that infertility is typically diagnosed after one year, and the majority of participants were convinced that it is less than 12 months or over 3 years. In the study of Ikimalo et al. [19], 32% of respondents provided the correct answer to the above question, whereas in the study of Iliyasu et al. [20], only 18.1% of the surveyed were able to accurately define infertility. These are interesting differences, most likely due to differences in the study populations. It is worrying that 40.5% of the surveyed women believed that 70% of the problem originates in women.

Women struggling with infertility often have low self-esteem, feel anger, despair, and jealousy toward other couples with a child, and experience constant anxiety. In contrast, in the study of Ali et al. [18], 40% of respondents answered that both men and women are responsible for infertility problems, while only 20% believed that infertility is related to men and 25% believed it is related to woman. Slightly over 40% of the respondents in our survey knew that the natural fertility of a woman decreases after 35 years of age, whereas in the study of Sabarre et al. [16], half of the surveyed respondents assumed that female fertility decreases as late as after 40 years of age. A similar result was observed in the study of Hammarberg et al. [12], in which one in four persons was aware that the reproductive capability of a woman reduces after 35 years of age. On the other hand, in the study of Chan et al. [10], among students, only 16% provided correct responses. Sorensen et al. [21] observed that young people want to first complete their education, find a stable partner, and advance in their career, which may have an impact on their reproductive health and fertility. Studies have shown that many women are not aware of the medical criteria for infertility, and as a result, they postpone their visit to a specialist, leading to a delayed diagnosis and treatment, thus reducing their chances of getting pregnant.

In total, 63.4% of the analyzed group of women demonstrated a good level of knowledge on the causes of infertility. Similar results were observed in the study of Ali et al. [18], in which the main factors indicated were ovulation disturbances (85%), tubal blockage (94%), and stress (65%). In the study of Abolfotouh et al. [22], the participants correctly identified most of the causes of infertility. However, additional factors mentioned by participants were supernatural forces. The respondents were from Saudi Arabia, and erroneous perceptions of infertility, including black magic (67.5%) and genies/supernatural causes (58.8%), were common among them. In the study of Iliyasu et al. [20], the respondents originated from Nigeria, and they believed that paranormal events were the main cause of infertility (92.1%). A small proportion of respondents mentioned salpingitis (27.7%) and irregular menstrual periods (24.6%) as the causes of infertility. The differences in the results observed in the above studies may be linked to differences in culture and in the content of sexual education programs.

In the study of Ali et al. [18], 74% of the surveyed indicated that infections of the female genital tract are among the possible causes of infertility. In the group analyzed by us, a majority of the participants (81.1%) believed that the presence of viral, bacterial, and infectious diseases should be checked during the evaluation for infertility. On the other hand, 28.8% of the respondents could not indicate any disease that could affect fertility. The respondents were aware that certain tests should be performed, but they had insufficient knowledge on the microorganisms that could contribute to limited fertility. This points out the urgent need for education on clinical symptoms of sexual organ infections and sexually transmitted diseases and the methods for preventing them. Untreated sexually transmitted diseases are among the major global causes of infertility in women [23].

In the study of Bunting et al. [24], the knowledge on fertility was found to vary depending on sociodemographic variables (e.g., education, employment, country’s development index). Variables related to the search for help had an influence on the knowledge about infertility, whereas those related to experiences in treating infertility or parenthood did not have any effect. These results show that the knowledge on reproductive health is mainly linked to education and not personal experiences [24]. In the present study, the level of knowledge on infertility diagnostics does not depend on the age of the surveyed women, nor on their educational level. Daniluk et al. [25] investigated the influence of sociodemographic factors, such as age, education level, and household income, on the level of knowledge about infertility. Their study showed that the educational level of the respondents had an effect on their knowledge of infertility, but it was not evident (r = 0.294). The correlations of the remaining variables were found to be low.

In the present study, the knowledge level on infertility diagnostics did not depend on the personal experiences of respondents in this field. Regardless of the experience, a majority of the respondents who had already been treated for infertility (45%) and those who had not been treated (61.5%) demonstrated an insufficient level of knowledge on infertility. In contrast, in the study of Abolfotouh et al. [22], women who had been or were treated for infertility during the study had more knowledge. Similar to the present study, in the study of Deatsman et al. [26], women who had been previously pregnant or had been treated for fertility did not have the expected higher level of knowledge. In summary, the knowledge on reproductive health has remained low over the years.

Limitations of the study include the non-validated questionnaire and sample size.

## 5. Conclusions

Information on reproductive health aspects should be appropriately disseminated through corresponding social campaigns, tailored to the age of the addressees. Educational programs are needed to promote the awareness of infertility and change the attitude of people of all ages toward this problem.

## Figures and Tables

**Table 1:** Characteristics of the study group.

Variable	Subgroup	n	%
Education	Primary	7	6.3%
	Professional	8	7.2%
	Secondary	49	44.1%
	Tertiary	47	42.3%
Place of residence	City < 20 thousand	21	18.9%
	City 20–100 thousand	19	9.0%
	City 100 to 500 thousand	10	17.1%
	City > 500 thousand	26	23.4%
	Village	35	31.5%
Marital status	In a relationship	33	29.7%
	Not in a relationship	78	70.3%
Age	<25	48	43.8%
	26–35	34	30.6%
	36–45	17	15.3%
	>45	12	10.8%
Professional status	Employed	76	68.5%
	Unemployed	35	31.5%
Progeny	Yes	85	76.6%
	No	26	23.4%
Trying to have a baby	Yes	90	81.1%
	No	21	19.9%
Infertility treatment	Yes	91	82.0%
	No	20	18.0%

**Table 2:** Responses of the respondents to individual questions.

Variable	Subgroup	n	%
What do you think is the period of regular intercourses after which we can speak about infertility?	<3 months	4	3.6%
	3–6 months	9	8.1%
	6 months to 1 year	38	34.1%
	>1 year	60	54.1%
Does a woman’s fertility depend on her age?	It does not depend	15	13.5%
	I do not know	8	7.2%
	Yes	40	36.0%
	Yes, it decreases after 35 years of age	48	43.2%
What do you think is the percentage distribution of infertility between women and men?	70% men, 30% women	6	5.4%
	50% men, 50% women	23	20.7%
	40% women, 45% men, 15% both	37	33.3%
	30% men, 70% women	45	40.5%
Do you think that testing for the presence of viral, bacterial, and infectious diseases should be performed during evaluation for infertility?	No	21	18.9%
	Yes	91	81.1%
Which of the mentioned diseases can be the cause of infertility in women?	Anatomical defects	64	57.7%
	Thyroid dysfunctions	86	77.5%
	Hormonal disorders	102	91.9%
	Polycystic ovary syndrome	95	85.6%
	Endometriosis	71	64.0%
	Obesity	65	58.6%
	Stress	78	70.3%
	Sudden gain or loss of body mass	46	41.4%
	Excessive physical effort	33	29.7%
What microbe-borne diseases may contribute to possessing the healthy baby?	HIV	39	35.1%
	*Treponema pallidum* bacteria (syphilis)	42	37.8%
	Chlamydia trachomatis	40	36.0%
	Cytomegalovirus	25	22.5%
	Rubella	41	36.9%
	Toxoplasmosis	43	43
	I do not know	32	28.8%
Does the human psyche have a significant influence on fertilization success?	Yes	93	83.8%
	No	2	1.8%
	I do not know	16	14.4%
If you experienced problems with infertility, where would you seek help or what would you do?	Visit to a family doctor	9	8.1%
	Visit to a gynecologist	104	93.7%
	Visit to a different physician, e.g., endocrinologist	84	75.7%
	Change to the lifestyle—giving up smoking	70	63.1%
	Being subject to pharmacotherapy	62	55.9%
	Adoption of a child	22	19.8%
	Other	1	0.9%

**Table 3:** Knowledge of the respondents on the causes of infertility.

Variable: Level of Knowledge on Causes of Infertility	n	%	Test Result
Insufficient (0–50)	44	39.64%	*χ*^2^ = 4.765 df = 1 *p* = 0.029
Rather good (51–66)	41	36.94%	
Good (67–80)	13	11.71%	
More than good (81–87)	7	6.31%	
Very good (88–100)	6	5.41%	

**Table 4:** Knowledge of the respondents on the symptoms that may accompany the diseases linked to limited fertility.

Variable: Level of Knowledge on Symptoms Related to Limited Fertility	n	%	Test Result
Insufficient (0–50)	65	58.6	*χ*^2^ = 3.252 df = 1 *p* = 0.071
Rather good (51–66)	36	32.4	
Good (67–80)	9	8.1	
Very good (88–100)	1	0.9	
Total	111	100.0	

## Data Availability

The data that support the findings of this study are available on request from the corresponding author.
